# Clinical Holistic Medicine: Holistic Sexology and Treatment of Vulvodynia Through Existential Therapy and Acceptance Through Touch

**DOI:** 10.1100/tsw.2004.115

**Published:** 2004-08-04

**Authors:** Søren Ventegodt, Mohammed Morad, Eytan Hyam, Joav Merrick

**Affiliations:** ^1^ The Quality of Life Research Center, Teglgårdstræde 4-8, DK-1452 Copenhagen K, Denmark; ^2^ Division of Community Health, Ben Gurion University, Beer-Sheva, Israel; ^3^ Soroka University Medical Center, Clalit Health Services, Faculty of Health Sciences, Ben Gurion University, Beer-Sheva, Israel; ^4^ National Institute of Child Health and Human Development, Office of the Medical Director, Division for Mental Retardation, Ministry of Social Affairs, Jerusalem and Zusman Child Development Center, Division of Pediatrics and Community Health, Ben Gurion University, Beer-Sheva, Israel

**Keywords:** quality of life, QOL, philosophy, human development, holistic medicine, public health, holistic health, holistic process theory, life mission theory, vulvodynia, vaginism, sexuality, ethics, Denmark

## Abstract

Sexual problems are found in four major forms: lack of libido, lack of arousal and potency, pain and discomfort during intercourse, and lack of orgasm. It is possible to work with a holistic approach to sexology in the clinic in order to find and repair the negative beliefs, repressions of love, and lack of purpose of life, which are the core to problems like arousal, potency, and pain with repression of gender and sexuality. It is important not to focus only on the gender and genitals in understanding the patient's sexual problems. It is of equal importance not to neglect the body, its parts, and the feelings and emotions connected to them. Shame, guilt, helplessness, fear, disgust, anger, hatred, and other strong feelings are almost always an important part of a sexual problem and these feelings are often “held” by the tissue of the pelvis and sexual organs. The patient with sexual problems can be helped both by healing existence in general and by discharging old painful emotions from the tissues. The later process of local healing is often facilitated by a simple technique: accepting contact via touch. This is a very simple technique, where the self-acceptance of the patient is to be promoted, for example, asking the female patient to put her hand on her stomach (uterus) or vulva, after which the holistic physician puts his hand supportively around hers. When done with care and after obtaining the necessary trust of the patient, this aspect of holding often releases the old negative emotions of shame bound to the touched areas. Afterwards, the emotional problems become a subject for conversational therapy and further holistic processing. Primary vulvodynia seems to be one of the diseases that can be cured after only a few successful sessions of working with acceptance through touch. The technique can be used as an isolated procedure or as a part of a pelvic examination. When touching the genitals with the intention of sexual healing, a written therapeutic contract with the patient is highly recommended and a strict ethical code is necessary to avoid malpractice. As about one woman in three suffers from sexual problems, many of which seemingly can be efficiently alleviated by the simple holistic techniques of “holding and processing”, it is very important that the holistic physician is also trained to work in the sexual sphere in order to be able to support his patients fully.

## INTRODUCTION

Sexology is the medical specialty concerned with sexual dysfunctions. The major breakthrough in this field was made by Masters and Johnson in the middle of the last century with the mapping of the human sexual functions and dysfunctions[1,2]. When a sexual problem cannot be solved together with the physician in his practice, the patient is sometimes referred to a sexologist, still using the techniques developed by these pioneers. As many people have sexual problems, it is not possible to refer everyone. Most problems should therefore be treated in general practice and most likely solved by conversation. There remains a residual quantity that apparently cannot be "talked away"[2], which is the subject of this paper.

It appears as though some children have developed a sexuality that is greatly impaired and destroyed by the lack of sexual acceptance, condemnation or merely failure in physical contact, because a child needs accepting touch[2,3], which of course must not lead to sexual abuse of the child. Intimacy is not sexuality, as every parent will know. Accepting the child's sexuality is not the same as encouraging sexual activity, but just acknowledging it as a sexual pole, either male or female[3,4,5].

The sexual problems resistant to conversational therapy are typically problems with acceptance of one's own sex and sexuality, which as originally suggested by Masters and Johnson can be a result of not having received the loving acceptance and touch needed in childhood[2,6]. If one is a girl, there is a need for her father to think she is lovely, delightful and "good enough to eat"[6]. It is obviously important that borders are not violated, but it is just as important that the father does not withdraw from physical contact, as he may for example, if he is afraid of his own sexual feelings or if he has repressed his own sexuality, so that he does not feel any physical interest in his daughter at all. The same applies to mother and son. Most parents show their acceptance or lack of acceptance through closeness and physical contact - ranging from warm, nourishing care to mental and physical violence. Regardless how good one is at talking, the conversation does not (at least according to our clinical experience) reach as deep as touch.

In Denmark today the common understanding is that repressing a child's sexual activity can be traumatising, while in other cultures and especially in the past child sexual activity was not allowed and such behaviour were seen as abnormal. At the times of Sigmund Freud children were send to the physician for masturbatory tendencies, but as times goes by and the subject has been studied scientifically, child sexuality has been more and more accepted as a normal aspect of child development[7].

As adults, a repressed childhood sexuality, can be observed in physical behaviour, where the person does not act with a sexual character. Either the person is sexless, or the person concerned is virtually of the opposite sex - too masculine, too feminine. That poses many problems to many people, who experience not having sufficient sexual attraction, not being sexually delightful, being sexually inadequate – e.g. lacking physical or orgasmic potency - and not having the desire for sex. Sexual problems seem in general to be related to poor quality of life[8].

Repression of sex and sexuality appears to happen through a decision that sex is wrong and shameful, or that one is not as delightful, as feminine or masculine as one ought to be. Early in life denial of one's sex can be done very effectively by deciding that one is of the opposite sex, in order maybe to meet the wisher of the parents, as we shall see in an example below. Based on our clinical observations, this results in some strangely unmanageable sexual problems, which are difficult to understand.

Existential healing or healing of the wholeness of the person on the deepest level of his or her existence is needed for the "abstract" sexual problems not related to a concrete physical problem. Before we continue, let us take a look on holistic medicine and the concept of existential healing.

## THE BASIS FOR CLINICAL HOLISTIC MEDICINE

The life mission theory[9,10,11,12,13] is based on the philosophy that everybody has a purpose of life or talents. Happiness comes from living this purpose and succeeding in expressing the core talent in life. To do this, it is important to develop as a person into what is know as the natural condition or a condition, where the person knows himself and uses all his efforts to achieve what is most important for him. The theory of talent[12] states that we have three major talents in life, called purpose, consciousness, and gender. In relation to this paper these dimensions may simply be: love, power, and sex. Gender and sexuality is a fundamental dimension of human existence, which must be in a sound, natural and un-denied state for the person to live and function naturally and in full power.

The holistic process theory of healing[14,15] and the related theories for salutogenesis[16,17], meaning of life[18], and quality of life [19,20,21] state that a return to the natural state of being is possible, whenever the person gets the resources needed for the existential healing. The resources needed are holding in the dimensions: awareness, respect, care, acknowledgment and acceptance with and support and processing in the dimensions: feeling, understanding and letting go of negative attitudes and beliefs. The preconditions for the holistic healing to take place are trust together with the intention of the healing taking place. Existential healing is not a local healing of any tissue, but a healing of the wholeness of the person, making him much more resourceful, loving and aware of himself, his own needs and wishes. In letting go of negative attitudes and beliefs the person returns to a more responsible existential position with an improved quality of life. The philosophical change taking place, when the person is healing is often a change towards preferring difficult problems and challenges, instead of avoiding difficulties in life[22,23,24,25,26,27,28,29]. The person who becomes happier and more resourceful is often also becoming more healthy, more talented and able of functioning[30,31,32].

Sexual problems are found in four major forms: lack of libido, lack of arousal and potency, pain and discomfort during intercourse and lack of orgasm[2]. It is possible to work with a holistic approach towards sexology in the clinic in order to find and repair the negative beliefs, repressions of love and lack of purpose of life, which seemingly are the core to problems like arousal, potency and pain, with repression of gender and sexuality[33,34]. The theory of talent[12] thus seems to be relevant for understanding human sexuality. It is highly important not to focus on the gender and genitals in understanding the patient's sexual problems, because many problems related to sex can be solved on the level of the whole person[2,33,34]. But as important as it is not to focus there, it is also essential not to neglect the body and the feelings connected to it. Shame, guilt, helplessness, fear and other strong feelings are almost always an important part of a sexual problem[2].

## ACCEPTANCE THROUGH TOUCH

Acceptance is one quality of "holding" that is more related to the healing of human existence in the sexual dimension than others. Acceptance has to do with the biological fact that we were not rejected from the womb, even when we were not syngenic with our mother, a marvellous biological fact still scientifically unexplained. Acceptance in early life has to do with close physical contact and touch, where the child needs touch almost more than anything. Sometimes our needs for touch and acceptance of our body, energies and functions were not fulfilled in early life, which can give us severe problems accepting ourselves as adults[2,3,4,5]. One of the areas of existence most vulnerable to lack of acceptance seems from our clinical experiences to be our sexuality.

As physicians, we have discovered in our practice that some of the problems related to gender and sexuality can be tackled by a simple technique: accepting contact via touch. It is possible to extract this simple, but essential aspect of the holistic pelvic examination[34], where it is a central feature and use it outside the primary medical pelvic examination.

The following case is an example, where the patient did not reveal her actual problem from the beginning, although she had been circling around her sexual problems with shame and embarrassment from the first conversation. Once we got a hold on the actual problem, progress was quick. We applied a very simple sexological technique, where self-acceptance was to be promoted, by asking the patient to put her hand on her own reproductive organs with the physician having his hand supportively around hers. The position of the physician’s hand mirrors exactly the position of the patient’s hand, so that the vulva is only touched directly by the patient and indirectly by the physician. The applied pressure is adjusted to the situation to optimise the therapeutic effect as described by Marion Rosen[35]. The indication for using this procedure in the holistic medical clinic must always be the physicians understanding of the patient’s need for physical acceptance. An attending nurse will give “holding” to the patient.

The procedure needs to be performed according to ethical standards. The holistic sexological procedure is derived from the holistic existential therapy, which involves re-parenting, massage and bodywork, conversational therapy, philosophical training, healing of existence during spontaneous regression to painful life events (gestalts), and close intimacy without any sexual involvement. In psychology, psychiatry and existential psychotherapy[36,37], touch is often allowed, but a sufficient distance between therapist and client is always kept, all clothes kept on, and it is even recommended that the first name is not taken into use to keep the relationship as formal and correct as possible[38]. The reason for this distance is to create a safety zone that removes the danger of psychotherapy leading to sexual involvement. In the original Hippocratic medicine[39], as well as in modern holistic existential therapy such a safety zone is not possible, because of the simultaneous work with all dimensions of existence, from therapeutic touch[40] of the physical body, feelings and mind, to sexuality and spirituality. The fundamental rule has since Hippocrates been that the physician must control his behaviour in order not to abuse his patient. The patients in holistic existential therapy and holistic sexology are often chronically sick, and their situation is often pretty hopeless, as many of them have been dysfunctional and incurable for many years, or they are suffering from conditions for which there is no efficient biomedical cures or therapies. The primary purpose of the holistic existential therapy is therefore to improve quality of life and secondly to improve health and ability. The severe conditions of the patients and their chronicity is what ethically justify the much more direct, intimate and intense method of holistic existential therapy, which integrates many different therapeutic elements, and works on many levels of the patients existence and personality at the same time. Holistic sexology is holistic existential therapy taken into the domain of sexology. The general ethical rule is that everything that does not harm and in the end helps the patient is allowed. An important aspect of the therapy is that the physician must be creative in practice, because no patients are alike, and invent a new treatment for every patient, as Yalom has suggested[36,37]. To perform the sexological technique of acceptance through touch in the genital areas of the body, the holistic sexologist must be able to control not only his/her behaviour, but also his sexual excitement to avoid any danger of the therapeutic session turning into sexual activity; the necessary level of mastery of this art can only be obtained through training and supervision. After the case stories we will come back to this important issue of ethics.

**Female, aged 33 years with vaginismus:** The patient arrived by her own choosing to the clinic, presenting her vaginismus still not sufficiently cured after 16 years of consultations with both physicians and alternative therapists. As nothing else seemed to work we found it acceptable to offer her the experimental sexological treatment of “acceptance through touch”, to which she consented. The six first consultations were used to prepare her for the treatment.

Seventh conversation: The patient related that immediately when intercourse begins she experience pain. When she was 17 years old she tried “a thousand times” to have intercourse with her boyfriend, but was unable to do so. The physician diagnosed vaginismus, which she still suffers from, although today she is able to have intercourse, most of the time with only modest discomfort. EXERCISE for the patient: Do not accept him, until you really have desire. Caress in all other ways first. On the couch, we worked on serious chronic tensions in the part of the adductor brevis muscle [one of the femur adductors], which inserts on the pubic bone. Along the way, she related that when she was 14 years old, she would lie in bed masturbating for two and a half hours at a time; she was sure that she was the only one from school who did it. Her very strong desire was then suppressed so that she did not even feel desire during petting, until her boyfriend made her go and see the physician, when she was 17 years old. We talked about such strong enjoyment being a great talent, which must be administered consciously. It is a great gift, but induces great resentment if it is not controlled (= condemnation as cheap, a “tart” etc.). We worked on her shame, guilt and self-condemnation, which were very marked, and slowly the muscles loosened. [The authors would like to point out that encouragement of sexual activity at such ages is not allowed under laws in the United States. The age of consent is 18 years in Arizona, California, Delaware, Florida, Idaho, Maine, Massachusetts, N. Dakota, Oregon, Tennessee, Utah, Virginia, and Wisconsin and 17 years in Colorado, Illinois, Louisiana, Missouri, Nebraska, New Mexico, New York and Texas. In Denmark sexual debut at the age of 13 years is not uncommon, though not legal, but in spite of this a teenager can get contraceptives from his/her physician and the physician will treat his/her sexual problems in very much the same way as an adult. In many countries the practice is different, so please adjust your practice to the law and culture of your country].

Eighth conversation and sexological procedure: acceptance through touch. It is going really well for her – everyone notices that she is well. Had her period last Saturday - regularly now for the third time in a row 29/5 with normal amount of menstrual bleeding instead of blood “pouring out”. Was “dumped” just after the last session by her boyfriend, which was not much fun. She is advised to let go of the boyfriend. On the couch we worked on acceptance of her sex - her hand right down against the vulva, mine (SV) supportively on top. We discussed that perhaps her purpose in life was to bring joy and happiness – and that made her completely desperate and unhappy. If she could choose a talent, it would be to be leader of large gatherings. “It’s so unfair that I did not become a man”, she says. She related that her mother and father thought she was a boy and she was to have been called Peter. There was a terribly great charge at this point of the conversation, which was then released.

We can here see a very great effect of this extremely simple technique. The sudden, completely spontaneous recognition that she was to have been a boy, with the serious consequences this has had for her in the form of self-condemnation and suppression of her own sex. The technique is unusual, because there is direct focus on her own acceptance of her physical sex. Because of the sexual taboo, she has apparently never received this acceptance previously. We often see, as is also the case here, that menstrual periods become far more regular and there is far less bleeding, when the woman has her relationship with her genitals and her sexuality normalised. Menstrual pain can also disappear. These findings are in concordance with the old tantric tradition of sexual yoga[5].

The next case is about a female, who had seen the physician (SV) many times and slowly gained her confidence. This enabled him to come close to her and give acceptance of her female side. It is the same treatment as above, but taken a step further. Note that although this holistic treatment with a focus on contact is rather unusual from a traditional medical perspective the professional border is well defined and sharp. Instead of avoiding touch, the physician uses it as a therapeutic resource and a way of helping the patient. As usual, we use the principle of minimal intervention.

**Female, aged 30 years saying “sex is not me”:** Tenth quality-of-life (QOL) conversation: Has been very sore in the lower abdomen, corresponding to the ovaries, she herself thinks. The discharge is normal and white. She still finds it difficult to accept physical contact, touch and care from her boyfriend. She feels nauseous, if he kisses her when she is not in the mood for it. On the couch, we work on the problems in the lower abdomen in the form of muscle tension on the inside of the pelvis, probably the psoas muscle [the “loin” running from the inside of the vertebral column to the femur], mostly on the right side. Her pelvic area appeared to be more cohesive and less blocked, but there was still severe tensions corresponding to the spina iliaca anterior superior [the anterior, superior tips of the pelvis].

Eleventh conversation and sexological procedure: acceptance through touch. I (SV) tell her that she looks so beautiful, fine and sensitive and like a pure innocent consciousness, but at the same time she looks to a great extent completely dead. She reacts positively to this acknowledgment and to the statement of my subjective impression of her problem. We have agreed that today I will play the role of the good father she has never had. She lies on the couch crying, and I hold her and kiss her neck and tell her that she is the apple of my eye. [The technique of re-parenting can only be done, when the holistic physician allows him/herself to behave *as if* the patient was his/her little child; it is of cause extremely important that this is done with full consent and after making an explicit therapeutic written contract of re-parenting. The kiss in the neck with no sexual intention given completely relaxed and another therapist or nurse present cannot be taken as a sexual act and will not be experienced as such by a patient in such therapeutic setting. Please notice that working with this degree of intimacy requires an experienced holistic, existential therapist with another person present, and despite of all these precautions, this is still unacceptable in many countries]. We talk about what type she is: social, sexual or survival and it is in her judgement as though all her problems are concerned with gender and sex. I agree with this. We therefore agree to work on her acceptance of her own gender and her own sexuality through accepting touch: Supportive acceptance through contact. She first holds herself on the outside of the vulva (on the outside of her briefs), with my hand supporting around hers. Afterwards I place my hand on her vulva (outside her briefs), while she holds her hand on top of mine. The reason for this step was for this patient to confront and process the shame bound to her genitals. The physician’s hand did not move during the procedure, it was resting for as long as she needed to confront the repressed feelings, which was called forth by this procedure, allowing her to enter the first phase of the holistic process of healing[14]] If she holds harder, I hold harder; if she holds more softly, I hold more softly; if she lets go, I let go too. In that way she controls the session, according to her need for support. [The patient response to the therapy was spontaneous regression to a very early age, judged from the way she spoke, moved, and from her non-abstract pattern of thinking combined with the characteristic expression in her face indication the regression, where she seemingly needed more contact and support than could be done by the indirect touch of the vulva normally used. Touching the patient’s vulva when she is in deep regressive therapy does not call on any sexual reaction, but is reacted to in the same way as a baby react to touch. Of course this further step requires a holistic physician being able to discriminate carefully between intimacy and sexuality to be able to hold and respect the sexual borders of the patient]. Conversation: In the meantime we talk about how her boyfriend only wants to do it right for her sexually, while she only wants to do it right for him. She is reading a book about women's orgasms – mostly for his sake. And when they are together, from his side it is only about her desire. The relationship has completely gone off the rails in my opinion, but because she feels completely devoid of value, she cannot allow herself to feel any joy or desire at all. It is a Gordian knot. Here she hits the "wall", and sees far more clearly than before how ill she is and what has to be done to cure her. She sobs inconsolably, lies on her side and asks me to hold her.

Twelfth conversation and sexological procedure repeated. Since last time: things have gone very well, success at work by working for her own sake, organised a family birthday etc. … She has been really like a teenager. We talk about her still being developed psychosexually like a big child. Sexually she made her breakthrough with her boyfriend last Friday and has since felt blissful. First vaginal orgasm together with him and first orgasm during intercourse. The feeling spread first to the whole vagina, then to the whole pelvis, then up into the abdomen and down into the thighs. On the couch, sexological procedure as last time is repeated. All in all, the patient is making fine progress and today looks like a really delightful woman. Confrontation in front of the mirror reveals that she hates the appearance of her own labia – shame and guilt. We must continue working on that.

## VULVODYNIA AND ACCEPTANCE THROUGH TOUCH/HOLISTIC GYNECOLOGY

Vulvodynia is a condition of unexplained chronic vulvar-vestibular pain with the etiology extremely illusive[41]. It is important to underline that vulvodynia can be primary vulvar discomfort, or secondary to a wide range of dermatological diseases, vulvar infections, inflammation, vulvar cancer and vulvar dysplasia[42], so it is of extreme importance to examine the patient for such an often hidden etiology, before giving the patient the diagnosis "primary vulvodynia". A study including 4,915 American woman aged 18 to 64 years showed that 16% of the women had experienced vulvodynia that lasted for at least three months and 7% had it at the time of the survey[43].

As it is known to be much more prevalent with the young adult woman[44] with a prevalence of 7%, making vulvodynia one of the most common hidden problems for young women. Only half consult a physician and the condition is very often misdiagnosed[45], but even with the correct diagnosis a variety of treatments are used, like muscle relaxing training, surgery, electric stimulation, biofeedback therapy, [46], tricylic antidepressants [46,47], topical nitroglycerin[48], steroids[49] or spinal cord stimulation [50]. Only about half the women got more than half the pain relieved[43] and that at the very best clinics, making the problem a vast unsolved problem for 5-10% of young woman. Often the chronic pains end in surgical procedures, giving some of the patients an immediate relief in their vulvar discomfort[51], but also giving many of the girls severe side effects like scaring and mutilating the vulva. CO2-laser treatment is sometimes used, but seems often to give scaring and severe mucosal atrophy[52]. One sad fact is that while most women with or without treatment will feel less troubled by vulvodynia over time, but most of the women will not experience, what deserves to be called "a cure"[43].

Interestingly, work with the pelvic floor muscle using electromyography-assisted rehabilitation seems effective in many cases of vulvodynia[53], illustrating the complex, presumably highly psychosomatic, dynamic of the sensations of the vulva and the whole pelvic region. Vulvodynia seems also to be correlated to QOL (quality of life)[54,55], shame seems to be highly correlated to vulvodynia[56], and shame and self-condemnation is exactly what the holistic procedure of acceptance through touch is intended to heal. Acceptance through touch, used alone or as a part of the holistic pelvic examination[34] seems to be an alternative strategy to alleviate the problem.

**Female, aged 24 years with primary vulvodynia:** Holistic gynecology. Known with /primary vulvodynia/ and sharp pain, when touching the vulva and introitus, as well as pains when inserting a finger into the vagina. Has always felt very uncomfortable, when touched, especially if the man uses a rubbing movement from the vagina towards the clitoris. Cannot touch herself with her fingers without being in pain and feeling uncomfortable. She thus never masturbates using her hand, but uses a teddy bear or another soft object. We discuss that she is generally very inhibited sexually and she would like to do something about that. /Sexual abuse?/ Slow gynaecological procedure following therapy contract[34]. Vulva, vagina in natural condition. Last menstruation took 7 days, no PMS. Due to the pain, no instruments in vagina. Exploration for tenderness, which gradually wears off through the session. We work with the painful areas, which send

> the patient into a deep feeling of humiliation, an unbearable feeling of shame and helplessness, a feeling of being held down and not being able to escape. As she works through the feelings, the pains in vulva-vagina disappear and at the end the patient can touch herself without further problems and feel good about caressing herself. A two-hour session well completed. EXCERCISE: masturbate using your hand; give yourself room to experience everything difficult associated with it. Write down what pops up and let us talk about it next time.

The problems of this patient did not come back. She described that it felt as if her vulva was "completely reorganised" during the session and after the session she noted that it now felt as an integrated part of her body for the first time in her life. She reported in the next session that she had no problems doing the exercise and that she was convinced that her vulvodynia was cured.

## DISCUSSION WITH ETHICAL CONSIDERATIONS

The holistic process of healing starts with the physician "caring for his patients". This care or maybe, in other words, professional love invites the trust of the patient. Treatment or "holding" that should result in a process of healing can only take place, when the patient fully trusts his or her physician. Holistic healing is not so much a technique, but rather a gift of caring in an unselfish support of the patients. Touching the genitals of a patient in the intention of giving acceptance cannot be successfully accomplished without the combination of care and a high ethical standard. To say this very clearly, only the physician who has a heart and care can touch the patient for the sake of healing the patient. Without such loving care, confidence and skilful holding[12,33,34] the procedure will not work.

In holistic sexological work with patients, where the physician tries to be present as a human being, the physician often has qualms and concerns. We have been extremely cautious and conscientious, but we have been painfully aware of "being on thin ice", when breaking one of the toughest taboos in the medical world, namely sex. It is severely frowned upon to touch the female private parts, if it is not in connection with a pelvic examination. There was no real place for what we intuitively feel to be especially important, namely the natural touch. A holistic physician may hold his patient in the same way that a father or mother supports his or her child through care or touch and have physical contact with precisely the area that is affected by problems. This is also the case where the most sensitive and difficult areas of the body are concerned.

It gives pause for thought that there are alternative therapists who sell these services, for example in the form of vaginal acupressure, which is increasingly commonly practised and accepted in for example Denmark. Vaginal acupressors have made a living by massaging acupressure points in the vaginas of women, who typically suffer from diminished libido[57]. This should put our fine senses as therapists into perspective. The idea of vaginal acupressure makes sense, since it is a thorough and persistent repeated confrontation of all the points in the lower abdomen that normally carry the sexual blockages. People who offer these controversial services typically have roots in Indian yoga (tantra[5]) and not in western medical science.

It is clear that we are battling against our absolute professional fear of confronting sexual problems in society in general and in the entire health service in particular. We may conclude that when blocked or traumatised areas generally react positively to touch and the laying-on of hands, it is not so surprising that sexual areas do so too. As long as it is ensured that the patient is in full control and is not violated – and that the therapist does not have sex with the patient in any form (in other words does not seduce her or manipulate her into a sexual relationship, what we very strikingly call "professional incest" and otherwise refrain from any sexual behaviour in relation to the patient) - such a treatment can never be unethical. It is an important thing for a physician to be able to support his patients fully, including within the sexual sphere.

The subject of ethics has been of utmost importance to the physician since Hippocrates (460-377 BCE) and whenever the physician touches the patient the ethics of the action must be considered. The problem of touch is more of an ethical than a legal problem: Why do you touch the patient, what is the intention? If the intention is for the physician to enjoy his patient – what we do most of the time with people in private - we consider this unethical, even if this is just holding hands. As often pointed out in the writings of Hippocrates[39], the physician should have the healing of the patient as his sole focus, and if the intention of the physicians is wholehearted and rooted in deep medical expertise to heal the patient, his life and existence (and in this intention touch any part of the body including the genitals), this is ethical. This kind of expertise is the expertise of the experienced holistic physician, who can take his/her patient into the state of consciousness we know as the process of salutogenesis[16,17], or holistic existential healing[14,15]. We believe, as did Hippocrates, that the physician's ethics seem to be proportional with his results with his patients[39]. Only the clearest of intentions can bring us outstanding results.

Let us conclude by saying that as far as men are concerned, our experience from the clinic is that sexual problems are often more mental and psychological. It is our experience that the deep, existential conversation on its own is sufficient to solve most of the problems, which do not have a physical etiology. When this is said, it is likely that a small fraction of men only will be helped by an understanding partner, making good reason for the famous and somewhat controversial use of substitute partners in the sexological clinic[2,58].

We believe that the technique of acceptance through gentle and respectful touching, (including when necessary the direct touch of the genitals possible when combining the technique of therapeutic touch with the pelvic examination[34]), followed by the existential conversation and further processing is sufficient to induce the holistic healing of most patient in the sexual realm. The next logical step in our research is to take the holistic methods we have developed into controlled clinical testing, where we believe the square curve paradigm to be useful[59].

## CONCLUSION

Sexual problems are found in four major forms: lack of libido, lack of arousal and potency, pain and discomfort during intercourse and lack of orgasm. It is possible to work with a holistic approach to sexology in the clinic in order to find and repair the negative beliefs, repressions of love and lack of purpose of life, which are the core to problems like arousal, potency and pain with repression of gender and sexuality. The theory of talent[12] therefore seems relevant for understanding human sexuality.

Shame, guilt, helplessness, fear, disgust, anger, hatred and other strong feelings are almost always an important part of a sexual problem. These feelings are often directly connected to the tissue of the sexual organs and related areas of the body. In order to initiate the process of healing the patient in the existential aspects related to gender and sexuality, we have discovered that some patients are helped by a simple technique of accepting contact via touch. This is a very simple sexological technique, in which the patient's self-acceptance is to be promoted, by asking the patient to put her hand on her stomach (uterus) or vulva, after which the physician puts his hand supportively around hers. This often releases the emotions bound to the areas, making them a subject for conversational therapy and holistic processing. This can also be an integrated part of a pelvic examination, if the procedure for this is followed[34]. The way this process was discovered was actually the intentional use of acceptance during the gynaecological standard procedure, with the somewhat surprising observation of a result of sexual healing, as described in [34].

The ethical aspects in holistic sexology is of extreme importance[34,40]. As long as the physician loves and cares for his patient, gets the trust of the patient, gives holding flawlessly and as long as it is ensured that the patient is in full control and is not in any way violated, such a treatment can never be unethical. The physician must also follow the ethical rules of the country, where the practice is performed and many countries have restrictions to such a holistic practice. It is important to understand that this contact is not and shall not be a sexual contact, and the most important qualification of the physician trained in the bodyworks of holistic medicine is his/her ability to control his own intention and level of sexual excitement to ensure that this contact never turns into a sexual contact.

Many young women suffer from vulvodynia, a painful state with no biomedical cure, but in our experience we have alleviated or even cured this condition by discharging the shame from the sexual organs with existential holistic therapy and acceptance through touch. As one patient in three has some problem related to sex and gender[8] related to personal development[12] it is important for the holistic physician to be able to support his patients fully, including in the sexual sphere.

## ACKNOWLEDGEMENTS

This study was supported by grants from IMK Almene Fond. The quality of life research was originally approved by the Copenhagen Scientific Ethical Committee under number (KF)V.100.2123/91.
